# Delayed gastric emptying after robot-assisted pancreatoduodenectomy: the Transatlantic Robot Pancreas Consortium (TROPANC)

**DOI:** 10.1007/s00464-026-12727-3

**Published:** 2026-03-30

**Authors:** Julia E. Menso, Bram L. J. van den Broek, Maurice J. W. Zwart, Mahsoem Ali, Brady Campbell, Emile Farah, Sarah Hays, Geert Kazemier, Aram E. Rojas, Nikhil V. Tirukkovalur, Jin He, Melissa E. Hogg, I. Quintus Molenaar, Alessandro Paniccia, Patricio M. Polanco, Amer H. Zureikat, Herbert J. Zeh, Bas Groot Koerkamp, Marc G. Besselink

**Affiliations:** 1 Department of Surgery, Amsterdam UMC, Location University of Amsterdam, De Boelelaan 1117, Amsterdam, The Netherlands; 2 Cancer Center Amsterdam, Amsterdam, The Netherlands; 3 Department of Surgery, Erasmus MC, Rotterdam, The Netherlands; 4 Department of Surgery, Amsterdam UMC, Location Vrije Universiteit, Amsterdam, The Netherlands; 5 Department of Surgery, Johns Hopkins University School of Medicine, Baltimore, MD USA; 6 Department of Surgery, UT Southwestern Medical Center, Dallas, TX USA; 7 Department of Surgery, Northshore University Health System, Chicago, IL USA; 8 Department of Surgery, University of Pittsburgh Medical Center, Pittsburgh, PA USA; 9 Department of Surgery, Regional Academic Cancer Center Utrecht (RAKU)/UMC Utrecht, Utrecht, The Netherlands

**Keywords:** Robot-assisted pancreatoduodenectomy, Gastrojejunostomy, Delayed gastric emptying

## Abstract

**Background:**

Delayed gastric emptying (DGE) is the most common complication of robot-assisted pancreatoduodenectomy (RPD). Large differences exist in DGE rate between centers and it remains unclear to what extent these are associated with surgical technique. This study assessed differences in DGE rate after RPD and predictors for DGE, including gastrojejunostomy (GJ) technique.

**Methods:**

Binational, multicenter retrospective cohort study including patients undergoing RPD from seven centers in the United States of America (USA) and the Netherlands (NL) (2011–2023). Data were retrospectively obtained from prospectively maintained databases. Multivariable analysis determined predictors for DGE, including GJ technique. Primary outcomes were DGE (ISGPS grade B/C), primary DGE (i.e., no other abdominal complications), and secondary DGE.

**Results:**

Overall, 1,842 patients undergoing RPD were included (USA 1,342, NL 500). Conversion rate was 5.0%, median hospital stay 8 days (6–13), and in-hospital/30-day mortality 1.5%. The rate of DGE grade B/C was 14.8%, primary DGE 5.7% (relative 38.9%), and secondary DGE 9.0% (relative 61.1%). The rates of DGE grade B/C (10.4% vs 26.8%, p < 0.001) and secondary DGE (4.4% vs 21.7%, p < 0.001) were lower in USA compared to NL, whereas the rate of primary DGE was comparable (6.0% vs 5.1%, p = 0.481). Overall, 1,259 (68.6%) GJs were sutured and 576 (31.4%) stapled. Sutured GJ was associated with a higher rate of DGE grade B/C (adjusted risk 18% vs 9%, p < 0.001) and primary DGE (adjusted risk 7% vs 3%, p < 0.001) compared to stapled GJ.

**Conclusions:**

This binational multicenter study found that DGE following RPD is mostly secondary to other complications. The association of stapled GJ with lower DGE rates should be confirmed by randomized studies. The most effective strategy to reduce the rate of DGE after RPD would be to prevent the causal underlying complications, particularly POPF.

**Graphic Abstract:**

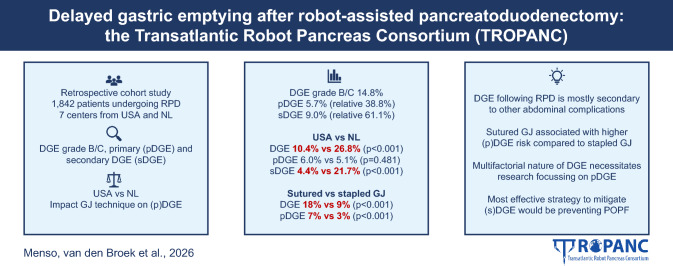

**Supplementary Information:**

The online version contains supplementary material available at 10.1007/s00464-026-12727-3.

Robot-assisted pancreatoduodenectomy (RPD) has developed rapidly over the past decades (1) aiming to enhance patient recovery compared to open pancreatoduodenectomy (OPD) (2–7). The implementation of RPD started in expert centers in the United States of America (USA) (8). Later, surgeons in the Netherlands (NL), with help from American colleagues, developed a training program which resulted in a 25% nationwide RPD implementation rate with currently over 1000 RPD performed (9).

Despite a highly standardized RPD surgical technique, the rates of morbidity and mortality following RPD vary widely globally (8–12). A 10–15% absolute difference in complication rates has been reported by the Global Audits on Pancreatic Surgery (GAPASURG) group, which includes the National Surgical Quality Improvement Program (NSQIP) and the nationwide audits from NL, Sweden, and Germany (13). One of the greatest differences is reported in the rate of DGE grade B/C with a 13% absolute difference between USA expert centers (20%) and a nationwide series (33%) (8, 9). After PD, DGE has a considerable impact on both hospital stay and healthcare costs (14, 15). Therefore, predictors for DGE may support timely initiation of preventive strategies. Differences in DGE rate may be explained by variations in surgical technique as the gastrojejunostomy (GJ) in RPD is less standardized compared to the pancreaticojejunostomy and hepaticojejunostomy.

Therefore, this study aimed to elucidate the observed variations in DGE rate following RPD by analyzing the impact of GJ technique. The hypothesis is that DGE (according to the ISGPS definition (16)) is mainly a secondary complication (secondary DGE), related to other abdominal complications, and that GJ technique is therefore not associated with DGE and primary DGE (pDGE).

## Methods

### Study design

A binational, retrospective multicenter cohort study including consecutive patients undergoing RPD in four centers in USA and three centers in NL (2011–2023). Data were collected from prospectively maintained databases in all centers and collated in a joint online database (Castor EDC, CIWIT B.V., Amsterdam, NL). The study was approved by the institutional review board of every participating center. This study followed the Strengthening the Reporting of Observational Studies in Epidemiology (STROBE) guidelines for reporting observational studies (Supplementary File 1) (17).

### Eligibility

All consecutive patients undergoing RPD for all indications in the TROPANC centers were included. Patients in whom RPD was converted to OPD were also included. Patient selection for RPD was according to each center's own protocol. Centers/surgeons who performed at least 150 RPD were eligible for TROPANC participation. The RPD surgeons in NL remained at their individual center from the beginning of their experience. As various RPD surgeons in USA moved to another center, their experience does not match the total center experience. Therefore, the required minimum of 150 RPD applied to surgeons and centers in NL and surgeons only in USA.

### Outcomes

The primary outcomes were the rate of DGE grade B/C according to the ISGPS definition (16), pDGE (defined as DGE without other abdominal complications), and secondary DGE (sDGE, defined as DGE with concomitant abdominal complications). Secondary outcomes included major complications (Clavien–Dindo grade ≥ 3), pancreas-specific complications, length of hospital stay, readmission, reoperation, and in-hospital/30-day mortality.

Baseline variables included age, sex, race, body mass index (BMI, kg/m^2^), preoperative pancreatitis and diabetes, preoperative biliary drainage, and neoadjuvant therapy. Intraoperative variables were conversion, operative time (min), blood loss (mL), pancreatic texture, main pancreatic duct (MPD) ≤ 3 mm, PJ technique (modified Blumgart versus other), procedure type (pylorus preservation versus resection), GJ technique (sutured versus stapled, intra- versus extracorporeal, and end-to-side versus side-to-side) and positioning (ante- versus retrocolic), bowel loop direction (biliary versus alimentary loop to the patient’s right), Roux-en-Y anastomosis, venous reconstruction, abdominal drain placement, and final diagnosis. Postoperative outcomes included major complication rate (Clavien–Dindo grade ≥ 3), postoperative pancreatic fistula (POPF) grade B/C (18), postpancreatectomy hemorrhage (PPH) grade B/C (19), bile leak grade B/C (20), DGE grade B/C (including pDGE and sDGE), length of hospital stay (days), readmission, reoperation, in-hospital/30-day mortality, and use of the PORSCH algorithm, which was implemented in 2018 in the Netherlands to improve detection of postoperative complications. This algorithm resulted in a lower threshold for imaging, increasing the number of reinterventions but reducing 90-day mortality (21). Additional data about standard local care, such as intervention technique details (e.g., GJ technique [pylorus preservation versus resection, sutured versus stapled, intra- versus extracorporeal, end-to-side versus side-to-side, ante- versus retrocolic, biliary versus alimentary loop towards the patient’s right] and abdominal drain[s] use) and postoperative management (e.g., postoperative feeding regimen) were collected with a surgeon’s survey and discussed in several online meetings. Six out of seven centers followed the same training program for RPD, increasing the homogeneity of GJ technique in this study (9). The follow-up was up to 30 days postoperatively.

### Statistical analysis

Three analyses were performed to assess baseline characteristics, intraoperative and postoperative outcomes in 1) the total cohort, 2) USA versus NL, and 3) sutured versus stapled GJ (post-hoc analysis of the strongest predictor for DGE grade B/C). Additionally, the rate of DGE grade B/C was assessed per ISGPS fistula risk category (22). Normally distributed continuous data were compared between groups with an unpaired T-test and presented as means with standard deviations (SDs). Non-normally distributed continuous data were analyzed with the non-parametric Kruskal–Wallis test and presented as medians with interquartile ranges (IQRs). Categorical data were analyzed with the chi-squared test and presented as frequencies with percentages.

Two sensitivity analyses were performed for the comparison between USA and NL 1) to assess the impact of disease diagnosis on the rate of DGE grade B/C and pDGE by excluding patients with final diagnosis pancreatic ductal adenocarcinoma (PDAC) and chronic pancreatitis, as these are associated with lower rates of POPF grade B/C due to fibrosis and obstruction (18) (i.e., leaving high-risk patients only), and 2) to assess the impact of the RPD learning curve on the rate of DGE grade B/C and pDGE by excluding the first 84 RPD per center (i.e., the mastery learning curve for textbook outcome) (23). Two centers were not adjusted for the learning curve as these surgeons had previously surpassed the learning curve in a different center.

Two separate multivariable logistic regression analyses were performed. First, a direct multivariable analysis identified independent predictors for DGE grade B/C and pDGE, while correcting for potential confounders: country/use of the PORSCH algorithm, age per 5 years, sex, BMI ≥ 35 kg/m^2^, preoperative pancreatitis and diabetes, preoperative biliary drainage, neoadjuvant therapy, final diagnosis PDAC and chronic pancreatitis, operative time (log-transformed), blood loss (log-transformed), pancreatic texture, MPD ≤ 3 mm, PJ technique, procedure type, GJ technique and positioning, bowel loop direction, Roux-en-Y anastomosis, venous reconstruction, use of abdominal drain(s), conversion (for DGE grade B/C and pDGE), POPF grade B/C, PPH grade B/C, and bile leak grade B/C (for DGE grade B/C).

Second, a direct multivariable analysis assessed the association of GJ technique (stapled versus sutured) on the risk of DGE grade B/C and pDGE, while correcting for potential confounders: country/use of the PORSCH algorithm, age per 5 years, sex, BMI ≥ 35 kg/m^2^, preoperative pancreatitis and diabetes, preoperative biliary drainage, neoadjuvant therapy, final diagnosis PDAC and chronic pancreatitis, pancreatic texture, MPD ≤ 3 mm, procedure type, GJ technique and positioning, bowel loop direction, Roux-en-Y anastomosis, and use of abdominal drain(s) (24). Regression standardization was used to estimate adjusted (marginal) risks and risk differences. Missing data were handled using multiple imputation (30 imputations and 50 iterations) and all parameter estimates were pooled across multiply imputed datasets using Rubin’s rules.

Data were analyzed with IBM SPSS Statistics version 28 for Windows and R, version 4.4.2, using the rms and marginal effects package (25). A two-tailed p value lower than 0.05 was considered to indicate statistical significance.

## Results

### Baseline characteristics

Overall, 1,842 patients undergoing RPD were included (USA n = 1,342 and NL n = 500). The median age was 68 years (IQR, 60–75) and 864 patients (46.9%) were female. Preoperative pancreatitis was found in 288 patients (15.9%) and diabetes in 472 patients (26.0%). Biliary drainage was performed in 1,055 patients (58.6%). Neoadjuvant chemo(radio)therapy was administered in 431 of 724 patients with PDAC (59.5%) (Table 1).

**Table 1:** Baseline characteristics

Baseline characteristics	Total (*n* = 1,842)	Sutured GJ (*n* = 1,259)	Stapled GJ (*n* = 576)	*P*-value
Age (years)	68 (60–75)	68 (60–75)	68 (60–75)	0.994
Female	864 (46.9%)	569 (45.2%)	292 (50.7%)	**0.028**
Race				** < 0.001**
White	1,522 (87.1%)	1,076 (89.7%)	440 (81.2%)	
African-American	127 (7.3%)	65 (5.4%)	61 (11.3%)	
Asian	48 (2.7%)	27 (2.3%)	21 (3.9%)	
Hispanic	29 (1.7%)	24 (2.0%)	5 (0.9%)	
Other	22 (1.3%)	7 (0.6%)	15 (2.8%)	
*Missing*	*94*	*60*	*34*	
BMI (kg/m^2^)	26.0 (23.1–29.6)	26.2 (23.1–29.8)	25.5 (22.9–29.1)	**0.033**
Preoperative pancreatitis	288 (15.9%)	219 (17.8%)	69 (12.0%)	**0.002**
*Missing*	*28*	*26*	*2*	
Preoperative diabetes	472 (26.0%)	332 (26.9%)	139 (24.2%)	0.233
*Missing*	*25*	*23*	*2*	
Preoperative biliary drainage	1,055 (58.6%)	763 (61.9%)	286 (51.1%)	** < 0.001**
*Missing*	*43*	*27*	*16*	
Neoadjuvant therapy*				** < 0.001**
Chemotherapy	370 (51.1%)	319 (25.5%)	95 (16.6%)	
Chemoradiation	61 (8.4%)	54 (4.3%)	10 (1.7%)	
*Missing*	*11*	*9*	*2*	
Surgery in center using the PORSCH algorithm	500 (27.1%)	313 (24.9%)	180 (31.3%)	**0.004**

Bold values are statistically significant

Values are presented as medians with interquartile ranges (IQR) and frequencies with percentages (%). BMI, body mass index; PDAC, pancreatic ductal adenocarcinoma. *Neoadjuvant therapy in patients with PDAC (n = 724, 518 sutured GJ versus 201 stapled GJ)

### Intraoperative outcome

The conversion rate was 5.0%. The median operative time was 389 min (332–465) and blood loss 180 mL (100–300). Soft pancreatic texture was found in 817 patients (50.0%) and MPD ≤ 3 mm in 936 patients (58.7%). Pylorus preservation was performed in 180 patients (9.8%). Overall, 1259 GJs (68.6%) were sutured and 576 (31.4%) stapled. Also, 1,544 GJs (91.3%) were created intracorporeally, 1,341 GJs (74.4%) with an end-to-side anastomosis, 1,692 GJs (100.0%) in an antecolic fashion, and 1,790 GJs (97.5%) with the alimentary loop to the patient’s right side. Only 10 patients (0.6%) received a Roux-en-Y anastomosis. Abdominal drain(s) were placed in 1,728 patients (94.6%). One center stopped using abdominal drain(s) in 2020. Overall, final pathology showed PDAC in 724 patients (39.4%) (Table 2).

**Table 2:** Intraoperative outcome

Intraoperative outcomes	Total (*n* = 1,842)	Sutured GJ (*n* = 1,259)	Stapled GJ (*n* = 576)	*P*-value
Conversion	92 (5.0%)	69 (5.5%)	23 (4.0%)	0.175
Operative time (min)	389 (332–465)	373 (324–439)	445 (387–522)	** < 0.001**
Blood loss (mL)	180 (100–300)	200 (100–350)	100 (50–200)	** < 0.001**
Soft pancreatic texture	817 (50.0%)	463 (40.8%)	353 (71.3%)	** < 0.001**
*Missing*	*208*	*125*	*81*	
MPD ≤ 3 mm	936 (58.7%)	580 (53.0%)	354 (71.5%)	** < 0.001**
* Missing*	*247*	*164*	*81*	
Modified Blumgart PJ	1,724 (93.6%)	1,238 (98.3%)	479 (83.2%)	** < 0.001**
Pylorus preservation	180 (9.8%)	180 (14.3%)	0 (0.0%)	** < 0.001**
*Missing*	*7*	*0*	*0*	
Sutured GJ	1,259 (68.6%)	NA	NA	NA
*Missing*	*7*			
Intracorporeal GJ	1,544 (91.3%)	1,080 (87.9%)	464 (100.0%)	** < 0.001**
*Missing*	*150*	*31*	*112*	
End-to-side GJ	1,341 (74.4%)	1,227 (100.0%)	114 (19.8%)	** < 0.001**
*Missing*	*39*	*32*	*0*	
Antecolic GJ	1,692 (100.0%)	1,228 (100.0%)	464 (100.0%)	*
*Missing*	*150*	*31*	*112*	
Alimentary loop to the right	1,790 (97.5%)	1,221 (97.0%)	569 (98.8%)	**0.020**
*Missing*	*7*	*0*	*0*	
Roux-en-Y anastomosis	10 (0.6%)	10 (0.8%)	0 (0.0%)	0.051
*Missing*	*150*	*31*	*112*	
Venous reconstruction	151 (8.5%)	132 (10.8%)	18 (3.2%)	** < 0.001**
Abdominal drain(s)	1,728 (94.6%)	1,175 (93.3%)	565 (98.1%)	** < 0.001**
Final pathology				** < 0.001**
No tumor	29 (1.6%)	14 (1.1%)	15 (2.6%)	
PDAC	724 (39.4%)	518 (41.2%)	201 (35.0%)	
Distal cholangiocarcinoma	181 (9.8%)	135 (10.7%)	45 (7.8%)	
Ampullary cancer	222 (12.1%)	170 (13.5%)	52 (9.1%)	
Duodenal adenocarcinoma	73 (4.0%)	52 (4.1%)	21 (3.7%)	
pNET	154 (8.4%)	92 (7.3%)	61 (10.6%)	
IPMN	245 (13.3%)	142 (11.3%)	103 (17.9%)	
SCN	13 (0.7%)	11 (0.9%)	2 (0.3%)	
SPN	18 (1.0%)	10 (0.8%)	8 (1.4%)	
Chronic pancreatitis	25 (1.4%)	21 (1.7%)	4 (0.7%)	
Duodenal ampullary adenoma	34 (1.8%)	25 (2.0%)	9 (1.6%)	
Other	120 (6.5%)	67 (5.3%)	53 (9.2%)	

Bold values are statistically significant

Values are presented as medians with interquartile ranges (IQR) and frequencies with percentages (%). GJ, gastrojejunostomy; MPD, main pancreatic duct; PJ, pancreaticojejunostomy; PDAC, pancreatic ductal adenocarcinoma; pNET, pancreatic neuroendocrine tumor; IPMN, intraductal papillary mucinous neoplasm; SCN, serous cyst neoplasm; SPN, solid pseudopapillary neoplasm; NA, not applicable. *No P-value could be generated

### Postoperative outcome

Overall, 270 patients (14.8%) had DGE grade B/C including 105 patients (5.7%, relative 38.9%) with pDGE (i.e., DGE without other abdominal complications) and 165 patients (9.0%, relative 61.1%) with sDGE (i.e., DGE with concomitant abdominal complications). The rates of DGE grade B/C (16.7% vs 10.9%, p = 0.001) and pDGE (6.9% vs 3.3%, p = 0.003) were higher in patients with sutured GJ compared to stapled GJ, whereas sDGE rate was comparable (9.8% vs 7.5, p = 0.123) (Table 3). The rate of DGE grade B/C increased in patients with ISGPS fistula risk category A to C (9.9% vs 14.1% vs 20.0%, p < 0.001). Regarding standard treatment, all centers started postoperatively with a liquid diet ranged for two to four days with thereafter a regular diet based on tolerability.

**Table 3:** Postoperative outcome

Postoperative outcomes	Total (*n* = 1,842)	Sutured GJ (*n* = 1,259)	Stapled GJ (*n* = 576)	*P*-value
Major complications (Clavien–Dindo grade ≥ 3)	589 (32.0%)	429 (34.1%)	156 (27.1%)	**0.003**
POPF grade B/C	279 (15.1%)	168 (13.3%)	111 (19.3%)	**0.001**
PPH grade B/C	125 (6.8%)	77 (6.1%)	46 (8.0%)	0.135
*Missing*	*1*	*0*	*1*	
Bile leak grade B/C	78 (4.2%)	32 (2.5%)	46 (8.0%)	** < 0.001**
*Missing*	*1*	*0*	*1*	
DGE grade B/C	270 (14.8%)	208 (16.7%)	62 (10.9%)	**0.001**
*Missing*	*15*	*10*	*5*	
Primary DGE	105 (5.7%)	86 (6.9%)	19 (3.3%)	**0.003**
Secondary DGE	165 (9.0%)	122 (9.8%)	43 (7.5%)	0.123
Hospital stay (days)	8 (6–13)	7 (6–12)	8 (6–14)	** < 0.001**
Readmission	434 (23.6%)	325 (25.8%)	108 (18.8%)	** < 0.001**
Reoperation	83 (4.5%)	58 (4.6%)	25 (4.3%)	0.799
In-hospital/30-day mortality	27 (1.5%)	17 (1.4%)	8 (1.4%)	0.947

Bold values are statistically significant

Values are presented as medians with interquartile ranges (IQR) and frequencies with percentages (%). GJ, gastrojejunostomy; POPF, postoperative pancreatic fistula; PPH, postpancreatectomy hemorrhage; DGE, delayed gastric emptying

In the total cohort, the rate of major complications was 32.0%, POPF 15.1%, PPH 6.8%, and bile leak 4.2%. The median length of hospital stay was 8 days (IQR, 6–13) with a readmission rate of 23.6%, reoperation 4.5%, and in-hospital/30-day mortality 1.5% (Table 3).

### DGE grade B/C

**Fig. 1:**
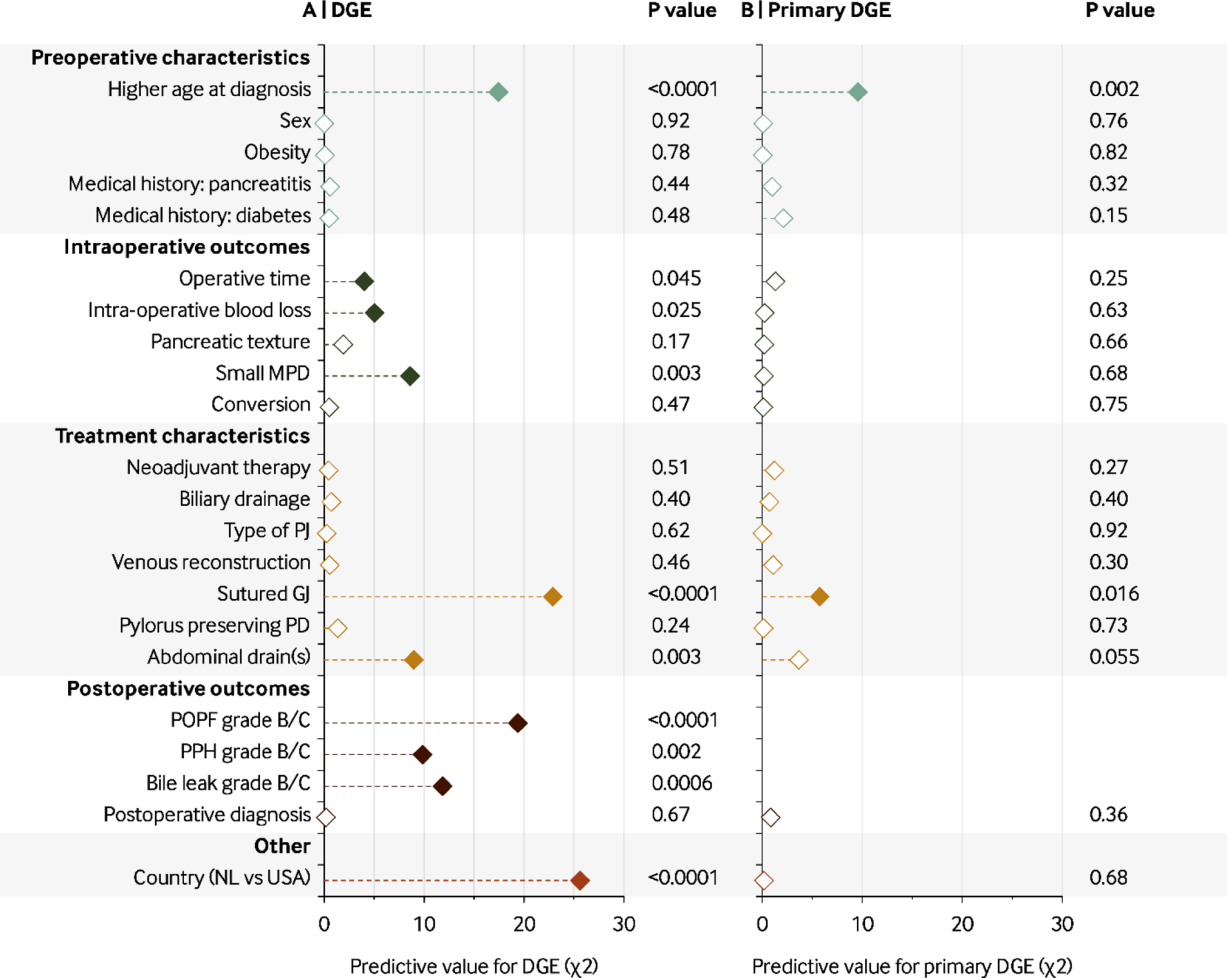
Predictors for DGE grade B/C and primary DGE following robot-assisted pancreatoduodenectomy. The Y-axis presents potential predictors for DGE grade B/C (**A**) and primary DGE (**B**). The X-axis represents the predictive value of the potential predictors. DGE, delayed gastric emptying; ASA. American Society of Anesthesiologists; MPD, main pancreatic duct; PJ, pancreaticojejunostomy; GJ, gastrojejunostomy; PD, pancreatoduodenectomy; POPF, postoperative pancreatic fistula; PPH, postpancreatectomy hemorrhage

The first multivariable analysis identified the following predictors for DGE grade B/C after RPD: age (OR per 5 years 1.16 [1.08–1.25], p < 0.001), operative time (OR 2.39 [1.02–5.57], p = 0.045), blood loss (OR 1.38 [1.05–1.82], p = 0.025), MPD ≤ 3 mm (OR 1.68 [1.18–2.37], p = 0.003), sutured GJ (OR 2.45 [1.67–3.59], p < 0.001), abdominal drain(s) (OR 2.64 [1.34–5.17], p = 0.003), POPF (OR 2.33 [1.62–3.36], p < 0.001), PPH (OR 2.16 [1.35–3.44], p = 0.002), and bile leak (OR 2.79 [1.59–4.89], p < 0.001) (Fig. 1 and Supplementary File 2). The second multivariable analysis showed that sutured GJ was associated with a higher risk of DGE grade B/C (adjusted risk 18% [95% CI: 16–21%] vs 9% [7–11%], absolute risk difference 10% [6–13%], p < 0.001) compared to stapled GJ (adjusted OR 2.50 [95% CI: 1.79–3.45], p < 0.001).

### Primary DGE

The first multivariable analysis identified the following predictors for pDGE after RPD: age (OR per 5 years 1.17 [1.05–1.30], p = 0.002) and sutured GJ (OR 1.99 [1.11–3.60], p = 0.016) (Fig. 1 and Supplementary File 3). The second multivariable analysis showed that sutured GJ was associated with a higher risk of pDGE (adjusted risk 7% [6–9%] vs 3% [2–5%], absolute risk difference 4% [2–6%], p < 0.001) compared to stapled GJ (adjusted OR 2.27 [1.33–4.00], p = 0.001).

### Outcomes USA vs NL

The rates of DGE grade B/C (10.4% vs 26.8%, p < 0.001) and sDGE (4.4% vs 21.7%, p < 0.001) were lower in USA compared to NL, whereas pDGE rate was comparable (6.0% vs 5.1%, p = 0.481). The rate of patients operated for PDAC was higher in USA (45.2% vs 23.8%, p < 0.001). Conversion rate was comparable between countries (4.8% vs 5.6%, p = 0.467). Fewer patients had a soft pancreatic texture in USA (44.4% vs 65.6%, p < 0.001) with a comparable rate of MPD ≤ 3 mm (57.7% vs 61.3%, p = 0.205). Sutured GJ (70.5% vs 63.5%, p = 0.004), pylorus preservation (13.4% vs 0.0%, p < 0.001), and abdominal drain(s) (99.7% vs 72.2%, p < 0.001) were more often used in USA (Supplementary File 4–6).

In patients with a high risk for POPF, i.e., after excluding patients with PDAC (n = 724, 39.4%) and chronic pancreatitis (n = 25, 1.4%), the rates of DGE grade B/C (11.2% vs 29.6%, p < 0.001) and sDGE (5.5% vs 24.9%, p < 0.001) remained lower in USA than NL, whereas pDGE rate remained comparable (5.7% vs 4.7%, p = 0.495) (Supplementary File 7).

Post-mastery learning curve, i.e., after excluding the first 84 RPD per center (26), the rates of DGE grade B/C (10.5% vs 23.8%, p < 0.001) and sDGE (4.4% vs 20.6%, p < 0.001) remained lower in USA than NL, whereas the pDGE rate remained comparable (6.2% vs 3.2%, p = 0.069) (Supplementary File 8). After the mastery learning curve, more patients were operated for PDAC and chronic pancreatitis in USA (45.7% vs 20.6%, p < 0.001).

## Discussion

This largest international series on RPD with 1,842 patients from seven expert centers in two countries found a 14.8% DGE grade B/C of which two-third was secondary to other complications. Major complications included 15.1% POPF, 6.8% PPH, and 4.2% bile leak, with 1.5% in-hospital/30-day mortality.

Three recent randomized controlled trials (RCTs) compared RPD with OPD and reported on DGE grade B/C. First, the EUROPA trial from Heidelberg (34%), second the trial from Beijing (11%), and third the European DIPLOMA-2 trial (24%). The EUROPA trial found more DGE grade B/C after RPD than OPD (34% vs 6%), whereas the other two RCTs found no significant differences (27–29). In contrast, single-center studies from Taipei, Taiwan, (409 RPD) and Baltimore, USA, (96 RPD) reported rates of DGE grade B/C as low as 4.4% and 5.2%, respectively (30). Few studies have reported on pDGE. A single-center study from Pisa, Italy, (50 RPD) reported a pDGE rate of 8% (31). A recent bi-center study from Italy, including 1,170 OPD, reported 13% DGE grade B/C and 3% pDGE (32), which is comparable to 6% pDGE in the current study.

Predictors for DGE grade B/C in this study were age, operative time, blood loss, MPD ≤ 3 mm, sutured GJ, abdominal drain(s), PPH, bile leak, and POPF. Among these, sutured GJ (OR 2.45, p < 0.001) and POPF (OR 2.33, p < 0.001) were the strongest predictors. A meta-analysis of 9,013 OPD and 192 RPD also identified age, blood loss, and MPD ≤ 3 mm as predictors for DGE with POPF (OR 2.09, p < 0.001), intra-abdominal collection (OR 3.58, p = 0.001), and abscess (OR 3.06, p < 0.001) as the strongest predictors (33). Although only one study in this systematic review included patients undergoing RPD (34), the high predictive value of POPF for DGE supports the hypothesis that DGE is mainly a secondary complication. Gastrojejunostomy technique and abdominal drainage were not included (33). A recent meta-analysis including 1,744 pancreatoduodenectomies from ten RCTs showed no impact of prophylactic abdominal drainage on DGE (35). Another meta-analysis of 18,961 pancreatoduodenectomies from 82 RCTs also could not identify an effective preventive strategy against DGE (14). Although one RCT including 137 pancreatoduodenectomies from nine centers in USA reported less DGE when no drains were used (9% vs 24%, p = 0.020), this trial was terminated prematurely due to a numerically higher mortality rate in the group without drainage (36).

Predictors for pDGE in the present study were age and sutured GJ. Sutured GJ was associated with a higher risk of pDGE compared to stapled GJ. This could be related to a larger anastomosis created by the stapled technique (34, 37). Larger GJ anastomoses and a vertical flow angle of less than 30 degrees in the alimentary loop have previously been associated with reduced DGE risk following RPD, but these findings did not address pDGE (34, 38). A single-center study from Pittsburgh with 192 patients undergoing RPD reported that sutured GJ was associated with a lower DGE rate compared to stapled GJ with a 45-mm or 60-mm stapler. The GJ size could have impacted their outcome as the same study reported that a greater GJ length was associated with less DGE (OR 0.53, p = 0.02) (34). The impact of GJ size could not be investigated in the present study as the surgeon’s survey showed that all centers used either a 60 mm stapler or sutured a GJ of 60-70 mm, although this GJ size is technically challenging in pylorus preserving PD. Other retrospective studies reported inconsistent findings regarding GJ technique (39–42). To date, no randomized trial has specifically addressed this issue. Given the observed absolute risk difference in DGE (10%) and pDGE (3%) after sutured versus stapled GJ, the clinical relevance of GJ technique remains to be confirmed. Pylorus preservation was not predictive for DGE in the present study, although this was performed in only 180 patients (9.8%) from one center and these duodenojejunostomies were sutured. One single-center RCT from Wakayama, Japan, including 130 OPD reported less DGE after pylorus resection compared to pylorus preservation, whereas the single-center RCTs from Heidelberg, Germany, and Kobe, Japan, showed no difference (43–45). Two RCTs from Japan showed less DGE with an antecolic position of the bowel loop (46, 47), whereas other RCTs showed no difference (48–50). The present study could not assess the impact of other GJ techniques and bowel loop positioning due to the low number of events in either one of the groups and missing data. For instance, no retrocolic GJs were performed, only 2.5% of GJs (two centers) positioned the alimentary loop to the patient’s left side, 8.7% of GJs (during the early learning curve of one center) were created extracorporeally (i.e., open surgery), and 0.6% of GJs included a Roux-en-Y anastomosis.

Consistent but unexplained differences were found between countries. The rates of DGE grade B/C (10.4% vs 26.8%) and sDGE (4.4% vs 21.7%) were lower in USA, without notable difference in pDGE (6.0% vs 5.1%). Also, major complications (23.8% vs 53.8%), POPF (8.3% vs 33.6%), PPH (5.7% vs 9.6%), bile leak (2.1% vs 10.0%), hospital stay (7 vs 11 days), reoperations (3.4% vs 7.4%), and in-hospital/30-day mortality (1.0% vs 2.6%) were better in USA. A clear explanation for these differences is currently lacking but some baseline imbalances could have contributed. Although more patients with preoperative pancreatitis/diabetes and final diagnosis PDAC/chronic pancreatitis were observed in USA, potentially lowering the risk of POPF and subsequently sDGE [18]. However, the sensitivity analysis excluding patients with PDAC and chronic pancreatitis still showed lower rates of DGE grade B/C and sDGE in USA than NL (p < 0.001) with a comparable pDGE rate (p = 0.495). Second, center-specific cohort sizes ranged from 105 to 683 patients and the proportion of RPD during the mastery learning curve from approximately 10% to 50% with very high-volume centers in USA compared to smaller volume centers in NL. Yet, the sensitivity analysis excluding the mastery learning curve still showed lower rates of DGE grade B/C and sDGE in USA than NL (p < 0.001) with a comparable pDGE rate (p = 0.069). A prolonged learning curve beyond 84 RPD may explain the difference in postoperative outcome between countries. Further research is needed to elucidate these discrepancies comprehensively as multiple factors (e.g., postoperative drain management (51)) are likely involved, many of which were beyond the scope of the current study. The GAPASURG collaboration previously reported considerable differences in patient outcome between Western registries of pancreatoduodenectomy. Although most postoperative outcomes were also in favor of USA, the rate of DGE grade B/C was 16.8% in USA compared to 18.9% in NL and no data were reported on pDGE (13). Notably, the relatively high complication rates in NL are consistent with those reported in the single-center EUROPA trial from Heidelberg, Germany, and the multicenter European DIPLOMA-2 trial with POPF rates of 38% and 23%, respectively (27, 29).

The results of this study should be interpreted considering some limitations. First, potential bias by interobserver variability was minimized by reclassifying all complications according to the ISGPS/ISGLS definitions (16, 18–20, 54). However, the current ISGPS definition for DGE does not provide strict criteria for the use of total parental nutrition and the interpretation of DGE onset, e.g., from the moment of dietary stagnation or absence of oral intake by postoperative day seven. This underscores the need for a more precise and standardized ISGPS definition of DGE. Additionally, the assessment of pancreatic texture during RPD is based on visual inspection rather than haptic feedback or objective measurements. Second, no data were available for preoperative diagnosis due to the retrospective study design. Therefore, the sensitivity analysis in patients with a high POPF risk was based on final pathology. Also, no data were available on smoking status, intraoperative fluid admission, pyloric dilation technique in pylorus-preserving RPD, and perioperative nasogastric tube management, enzyme use and nutritional counseling. Third, international differences in health care system may have impacted the results, such as missed readmissions. This was minimized by collecting data on follow-up. Besides, international differences in social determinants and health care accessibility (e.g., insurance coverage) could have influenced patient selection and medical decision making [46, 47]. Finally, although surgical techniques were highly standardized across centers and all patients were treated according to the enhanced recovery after surgery (ERAS) program (55), postoperative management protocols were not uniform. The PORSCH study led to a very low threshold for percutaneous drainage after pancreatoduodenectomy in NL. This approach likely resulted in a higher detection of postoperative complications, but it reduced postoperative mortality in NL, although mortality remained lowest in USA [48].

In conclusion, DGE grade B/C is mainly a secondary complication, related to other abdominal complications, mostly POPF. Stapled GJ was associated with less DGE and pDGE compared to sutured GJ, but its clinical impact should be interpreted carefully due to the multifactorial nature of DGE. Randomized studies on pDGE specifically are needed to confirm this finding. Finally, the high incidence of sDGE suggest that the most effective strategy to prevent the majority of DGE after RPD would be preventing other complications, particularly POPF.

## Supplementary Information

Below is the link to the electronic supplementary material.

Supplementary file1 (DOCX 111 KB)
