# Supplementary material for: Delayed gastric emptying after robot-assisted pancreatoduodenectomy: the Transatlantic Robot Pancreas Consortium (TROPANC)
Source: Surg Endosc. 2026 Mar 30;40(6):5176–86. doi: 10.1007/s00464-026-12727-3 (PMC13246809; doi:10.1007/s00464-026-12727-3)
Supplement: Supplementary file 1 — Supplementary file1 (DOCX 111 KB) [file 464_2026_12727_MOESM1_ESM.docx]

# Supplementary file 1: STROBE checklist

|  | Item No | Recommendation | Location where item is reported |
| --- | --- | --- | --- |
| **Title and abstract** | 1 | (*a*) Indicate the study’s design with a commonly used term in the title or the abstract | Page 1 |
|  |  | (*b*) Provide in the abstract an informative and balanced summary of what was done and what was found | Page 2 |
| Introduction | | | |
| Background/rationale | 2 | Explain the scientific background and rationale for the investigation being reported | Page 3 |
| Objectives | 3 | State specific objectives, including any prespecified hypotheses | Page 3 |
| Methods | | | |
| Study design | 4 | Present key elements of study design early in the paper | Page 4 |
| Setting | 5 | Describe the setting, locations, and relevant dates, including periods of recruitment, exposure, follow-up, and data collection | Page 4-5 |
| Participants | 6 | (*a*) Give the eligibility criteria, and the sources and methods of selection of participants. Describe methods of follow-up | Page 4 |
|  |  | (*b*) For matched studies, give matching criteria and number of exposed and unexposed | Not applicable |
| Variables | 7 | Clearly define all outcomes, exposures, predictors, potential confounders, and effect modifiers. Give diagnostic criteria, if applicable | Page 4-5 |
| Data sources/ measurement | 8 | For each variable of interest, give sources of data and details of methods of assessment (measurement). Describe comparability of assessment methods if there is more than one group | Page 5-7 |
| Bias | 9 | Describe any efforts to address potential sources of bias | Page 6-7 |
| Study size | 10 | Explain how the study size was arrived at | Page 4 |
| Quantitative variables | 11 | Explain how quantitative variables were handled in the analyses. If applicable, describe which groupings were chosen and why | Page 5-6 |
| Statistical methods | 12 | (*a*) Describe all statistical methods, including those used to control for confounding | Page 6-7 |
|  |  | (*b*) Describe any methods used to examine subgroups and interactions | Page 6 |
|  |  | (*c*) Explain how missing data were addressed | Page 7 |
|  |  | (*d*) If applicable, explain how loss to follow-up was addressed | Not applicable |
|  |  | (*e*) Describe any sensitivity analyses | Page 6 |
| Results | | | |
| Participants | 13 | (a) Report numbers of individuals at each stage of study—eg numbers potentially eligible, examined for eligibility, confirmed eligible, included in the study, completing follow-up, and analysed | Page 9 |
|  |  | (b) Give reasons for non-participation at each stage | Not applicable |
|  |  | (c) Consider use of a flow diagram | Not applicable |
| Descriptive data | 14 | (a) Give characteristics of study participants (eg demographic, clinical, social) and information on exposures and potential confounders | Page 8 |
|  |  | (b) Indicate number of participants with missing data for each variable of interest | Table 1-3  Supplements |
|  |  | (c) Summarise follow-up time (eg, average and total amount) | Not applicable |
| Outcome data | 15 | Report numbers of outcome events or summary measures over time | Page 8-10 |
| Main results | 16 | (*a*) Give unadjusted estimates and, if applicable, confounder-adjusted estimates and their precision (eg, 95% confidence interval). Make clear which confounders were adjusted for and why they were included | Page 8-10 |
|  |  | (*b*) Report category boundaries when continuous variables were categorized | Page 8-10 |
|  |  | (*c*) If relevant, consider translating estimates of relative risk into absolute risk for a meaningful time period | Not applicable |
| Other analyses | 17 | Report other analyses done—eg analyses of subgroups and interactions, and sensitivity analyses | Page 9-10 |
| Discussion | | | |
| Key results | 18 | Summarise key results with reference to study objectives | Page 11 |
| Limitations | 19 | Discuss limitations of the study, taking into account sources of potential bias or imprecision. Discuss both direction and magnitude of any potential bias | Page 14-15 |
| Interpretation | 20 | Give a cautious overall interpretation of results considering objectives, limitations, multiplicity of analyses, results from similar studies, and other relevant evidence | Page 11-14 |
| Generalisability | 21 | Discuss the generalisability (external validity) of the study results | Page 11-15 |
| Other information | | | |
| Funding | 22 | Give the source of funding and the role of the funders for the present study and, if applicable, for the original study on which the present article is based | Page 18 |

Legend supplementary file 1: Reference: Vandenbroucke, J.P., et al., *Strengthening the Reporting of Observational Studies in Epidemiology (STROBE): explanation and elaboration.* Int J Surg, 2014. **12**(12): p. 1500-24.

# Supplementary file 2: Predictors for DGE grade B/C following robot-assisted pancreatoduodenectomy

| **DGE grade B/C** | **Univariable analysis** | | **Multivariable analysis*** | |
| --- | --- | --- | --- | --- |
|  | **OR (95% CI)** | **P-value** | **OR (95% CI)** | **P-value** |
| Age per 5 years | 1.14 (1.07–1.21) | **<0.001** | 1.16 (1.08–1.25) | **<0.001** |
| Male | 1.22 (0.94–1.59) | 0.134 | 0.98 (0.73–1.32) | 0.918 |
| BMI <35 kg/m^2^ | 1.25 (0.73–2.17) | 0.407 | 0.92 (0.51–1.66) | 0.778 |
| No preoperative pancreatitis | 1.41 (0.95–2.08) | 0.088 | 1.19 (0.77–1.84) | 0.441 |
| No preoperative diabetes | 1.09 (0.80–1.47) | 0.598 | 0.89 (0.63–1.24) | 0.480 |
| No preoperative biliary drainage | 1.22 (0.93-1.59) | 0.136 | 1.14 (0.84–1.54) | 0.397 |
| Upfront surgery | 1.89 (1.35–2.63) | **<0.001** | 1.16 (0.75–1.79) | 0.509 |
| Other than PDAC/chronic pancreatitis | 1.70 (1.28–2.24) | **<0.001** | 0.92 (0.62–1.36) | 0.673 |
| Operative time (log-transformed) | 0.91 (0.48–1.71) | 0.757 | 2.39 (1.02–5.57) | **0.045** |
| Blood loss (log-transformed) | 1.45 (1.19–1.77) | **<0.001** | 1.38 (1.05–1.82) | **0.025** |
| Soft pancreatic texture | 1.81 (1.37–2.39) | **<0.001** | 1.29 (0.90–1.85) | 0.167 |
| MPD ≤3 mm | 1.59 (1.19–2.14) | **0.002** | 1.68 (1.18–2.37) | **0.003** |
| Modified Blumgart PJ | 1.53 (0.83–2.83) | 0.171 | 0.83 (0.41–1.70) | 0.615 |
| Pylorus resection | 1.09 (0.70–1.69) | 0.801 | 0.73 (0.43–1.23) | 0.243 |
| Sutured GJ | 1.64 (1.20–2.22) | **0.001** | 2.45 (1.67–3.59) | **<0.001** |
| Venous reconstruction | 1.01 (0.63–1.62) | 0.969 | 1.24 (0.71–2.18) | 0.458 |
| Abdominal drain(s) | 1.06 (0.59–1.89) | 0.854 | 2.64 (1.34–5.17) | **0.003** |
| Conversion | 1.35 (0.78–2.32) | 0.283 | 0.78 (0.40–1.55) | 0.471 |
| POPF grade B/C | 4.36 (3.25–5.84) | **<0.001** | 2.33 (1.62–3.36) | **<0.001** |
| PPH grade B/C | 3.14 (2.11–4.67) | **<0.001** | 2.16 (1.35–3.44) | **0.002** |
| Bile leak grade B/C | 4.68 (2.92–7.48) | **<0.001** | 2.79 (1.59–4.89) | **<0.001** |

Legend supplementary file 2: Values are presented as odds ratios (OR) with 95% confidence intervals (CI). DGE, delayed gastric emptying; BMI, body mass index; PDAC, pancreatic ductal adenocarcinoma; MPD, main pancreatic duct; PJ, pancreaticojejunostomy; POPF, postoperative pancreatic fistula; PPH, postpancreatectomy hemorrhage. *Corrected for country.

# Supplementary file 3: Predictors for primary DGE following robot-assisted pancreatoduodenectomy

| **Primary DGE** | **Univariable analysis** | | **Multivariable analysis*** | |
| --- | --- | --- | --- | --- |
|  | **OR (95% CI)** | **P-value** | **OR (95% CI)** | **P-value** |
| Age per 5 years | 1.19 (1.07–1.31) | **<0.001** | 1.17 (1.05–1.30) | **0.002** |
| Male | 1.14 (0.76–1.69) | 0.528 | 1.07 (0.70–1.62) | 0.758 |
| BMI <35 kg/m^2^ | 1.28 (0.55–2.94) | 0.563 | 1.11 (0.46–2.66) | 0.821 |
| No preoperative pancreatitis | 1.41 (0.76–2.56) | 0.282 | 1.37 (0.72–2.60) | 0.322 |
| Preoperative diabetes | 1.52 (0.99–2.33) | 0.057 | 1.39 (0.90–2.15) | 0.146 |
| No preoperative biliary drainage | 1.05 (0.69–1.59) | 0.821 | 1.21 (0.78–1.87) | 0.395 |
| Upfront surgery | 1.06 (0.68–1.67) | 0.802 | 1.38 (0.78–2.42) | 0.269 |
| PDAC/chronic pancreatitis | 1.19 (0.80–1.79) | 0.386 | 1.28 (0.77–2.18) | 0.359 |
| Operative time (log-transformed) | 0.40 (0.15–1.03) | 0.057 | 0.49 (0.15–1.65) | 0.250 |
| Blood loss (log-transformed) | 1.14 (0.84–1.56) | 0.387 | 1.11 (0.73–1.68) | 0.632 |
| Hard pancreatic texture | 1.27 (0.83–1.96) | 0.276 | 1.12 (0.68–1.85) | 0.665 |
| MPD >3 mm | 1.28 (0.83–1.96) | 0.257 | 0.90 (0.56–1.45) | 0.676 |
| Modified Blumgart PJ | 1.73 (0.63–4.79) | 0.290 | 1.06 (0.36–3.13) | 0.916 |
| Pylorus preservation | 1.43 (0.80–2.56) | 0.226 | 1.13 (0.57–2.21) | 0.734 |
| Sutured GJ | 2.13 (1.30–3.57) | **0.003** | 1.99 (1.11–3.60) | **0.016** |
| Venous reconstruction | 1.70 (0.93–3.13) | 0.087 | 1.45 (0.73–1.90) | 0.299 |
| Abdominal drain(s) | 3.07 (0.75–12.65) | 0.120 | 3.45 (0.78–15.2) | 0.055 |
| Conversion | 1.17 (0.50–2.74) | 0.722 | 0.86 (0.33–2.26) | 0.755 |

Legend supplementary file 3: Values are presented as odds ratios (OR) with 95% confidence intervals (CI). DGE, delayed gastric emptying; BMI, body mass index; PDAC, pancreatic ductal adenocarcinoma; MPD, main pancreatic duct; PJ, pancreaticojejunostomy. *Corrected for country.

# Supplementary file 4: Baseline characteristics in USA versus NL

| **Baseline characteristics** | **Total (n=1,842)** | **USA (n=1,342)** | **NL (n=500)** | **P-value** |
| --- | --- | --- | --- | --- |
| Age (years) | 68 (60–75) | 68 (60–74) | 69 (61–75) | 0.144 |
| Female | 864 (46.9%) | 640 (47.7%) | 224 (44.8%) | 0.269 |
| Race  White  African-American  Asian  Hispanic  Other  *Missing* | 1,522 (87.1%)  127 (7.3%)  48 (2.7%)  29 (1.7%)  22 (1.3%)  *94* | 1,116 (84.2%)  118 (8.9%)  42 (3.2%)  28 (2.1%)  21 (1.6%)  *17* | 406 (96.0%)  9 (2.1%)  6 (1.4%)  1 (0.2%)  1 (0.2%)  *77* | **<0.001** |
| BMI (kg/m^2^) | 26.0 (23.1–29.6) | 26.4 (23.3–30.3) | 24.9 (22.5–27.7) | **<0.001** |
| Preoperative pancreatitis  *Missing* | 288 (15.9%)  *28* | 229 (17.1%)  *1* | 59 (12.5%)  *27* | **0.019** |
| Preoperative diabetes  *Missing* | 472 (26.0%)  *25* | 371 (27.6%)  *0* | 101 (21.3%)  *25* | **0.006** |
| Preoperative biliary drainage  *Missing* | 1,055 (58.6%)  *43* | 806 (60.1%)  *2* | 249 (54.2%)  *41* | **0.027** |
| Neoadjuvant therapy*  Chemotherapy  Chemoradiation | 370 (51.1%)  61 (8.4%) | 350 (57.8%)  47 (7.8%) | 20 (16.9%)  14 (11.9%) | **<0.001** |
| Surgery in center using the PORSCH algorithm | 500 (27.1%) | 0 (0.0%) | 500 (100.0%) | **<0.001** |

Legend supplementary file 4: Values are presented as medians with interquartile ranges (IQR) and frequencies with percentages (%). USA, United States of America; NL, the Netherlands; BMI, body mass index. *Neoadjuvant therapy in patients with PDAC (n=724, 606 USA vs 118 NL).

# Supplementary file 5: Intraoperative outcome in USA versus NL

| **Intraoperative outcomes** | **Total (n=1,842)** | **USA (n=1,342)** | **NL (n=500)** | **P-value** |
| --- | --- | --- | --- | --- |
| Conversion | 92 (5.0%) | 64 (4.8%) | 28 (5.6%) | 0.467 |
| Operative time (min) | 389 (332–465) | 377 (325–440) | 420 (359–510) | **<0.001** |
| Blood loss (mL) | 180 (100–300) | 150 (90–300) | 200 (100–400) | **<0.001** |
| Soft pancreatic texture  *Missing* | 817 (50.0%)  *208* | 533 (44.4%)  *141* | 284 (65.6%)  *67* | **<0.001** |
| MPD ≤3 mm  *Missing* | 936 (58.7%)  *247* | 672 (57.7%)  *178* | 264 (61.3%)  *69* | 0.205 |
| Modified Blumgart PJ | 1,724 (93.6%) | 1,236 (92.1%) | 488 (97.6%) | **<0.001** |
| Pylorus preservation  *Missing* | 180 (9.8%)  *7* | 180 (13.4%)  *0* | 0 (0.0%)  *7* | **<0.001** |
| Sutured GJ  *Missing* | 1,259 (68.6%)  *7* | 946 (70.5%)  *0* | 313 (63.5%)  *7* | **0.004** |
| Intracorporeal GJ  *Missing* | 1,544 (91.3%)  *150* | 1,342 (100.0%)  *0* | 202 (57.7%)  *150* | **<0.001*** |
| End-to-side GJ  *Missing* | 1,341 (74.3%)  *38* | 948 (70.6%)  *0* | 393 (85.1%)  *38* | **<0.001** |
| Antecolic GJ  *Missing* | 1,692 (100.0%)  *150* | 1,342 (100.0%)  *0* | 350 (100.0%)  *150* | **<0.001*** |
| Alimentary loop to the right  *Missing* | 1,790 (97.5%)  *7* | 1,342 (100.0%)  *0* | 448 (90.9%)  *7* | **<0.001*** |
| Roux-en-Y anastomosis  *Missing* | 10 (0.6%)  *150* | 0 (0.0%)  *0* | 10 (2.9%)  *150* | **<0.001*** |
| Venous reconstruction | 151 (8.5%) | 127 (9.6%) | 24 (5.3%) | **0.005** |
| Abdominal drain(s) | 1,728 (94.6%) | 1,338 (99.7%) | 405 (81.1%) | **<0.001** |
| Final pathology  No tumor  PDAC  Distal cholangiocarcinoma  Ampullary cancer  Duodenal adenocarcinoma  pNET  IPMN  SCN  SPN  Chronic pancreatitis  Duodenal ampullary adenoma  Other | 29 (1.6%)  724 (39.4%)  181 (9.8%)  222 (12.1%)  73 (4.0%)  154 (8.4%)  245 (13.3%)  13 (0.7%)  18 (1.0%)  25 (1.4%)  34 (1.8%)  120 (6.5%) | 18 (1.3%)  606 (45.2%)  109 (8.1%)  136 (10.1%)  52 (3.9%)  107 (8.0%)  166 (12.4%)  8 (0.6%)  15 (1.1%)  14 (1.0%)  18 (1.3%)  93 (6.9%) | 11 (2.2%)  118 (23.8%)  72 (14.5%)  86 (17.3%)  21 (4.2%)  47 (9.5%)  79 (15.9%)  5 (1.0%)  3 (0.6%)  11 (2.2%)  16 (3.2%)  27 (5.4%) | **<0.001** |

Legend supplementary file 5: Values are presented as medians with interquartile ranges (IQR) and frequencies with percentages (%). USA, United States of America; NL, the Netherlands; MPD, main pancreatic duct; PJ, pancreaticojejunostomy; GJ, gastrojejunostomy; PDAC, pancreatic ductal adenocarcinoma; pNET, pancreatic neuroendocrine tumor; IPMN, intraductal papillary mucinous neoplasm; SCN, serous cyst neoplasm; SPN, solid pseudopapillary neoplasm. *Fisher’s exact test due to the low number of events.

# Supplementary file 6: Postoperative outcome in USA versus NL

| **Postoperative outcomes** | **Total (n=1,842)** | **USA (n=1,342)** | **NL (n=500)** | **P-value** |
| --- | --- | --- | --- | --- |
| Major complications  (Clavien–Dindo grade ≥3) | 589 (32.0%) | 320 (23.8%) | 269 (53.8%) | **<0.001** |
| POPF grade B/C | 279 (15.1%) | 111 (8.3%) | 168 (33.6%) | **<0.001** |
| PPH grade B/C  *Missing* | 125 (6.8%)  *1* | 77 (5.7%)  *1* | 48 (9.6%)  *0* | **0.003** |
| Bile leak grade B/C  *Missing* | 78 (4.2%)  *1* | 28 (2.1%)  *0* | 50 (10.0%)  *1* | **<0.001** |
| DGE grade B/C  *Missing* | 270 (14.8%)  *15* | 139 (10.4%)  *4* | 131 (26.8%)  *11* | **<0.001** |
| Primary DGE | 105 (5.7%) | 80 (6.0%) | 25 (5.1%) | 0.481 |
| Secondary DGE | 165 (9.0%) | 59 (4.4%) | 106 (21.7%) | **<0.001** |
| Hospital stay (days) | 8 (6–13) | 7 (6–10) | 11 (7–21) | **<0.001** |
| Readmission | 434 (23.6%) | 327 (24.4%) | 107 (21.4%) | 0.182 |
| Reoperation | 83 (4.5%) | 46 (3.4%) | 37 (7.4%) | **<0.001** |
| In-hospital/30-day mortality | 27 (1.5%) | 14 (1.0%) | 13 (2.6%) | **0.013** |

Legend supplementary file 6: Values are presented as medians with interquartile ranges (IQR) and frequencies with percentages (%). USA, United States of America; NL, the Netherlands; POPF, postoperative pancreatic fistula; PPH, postpancreatectomy hemorrhage; DGE, delayed gastric emptying.

# Supplementary file 7: Postoperative outcome in patients with other diagnoses than PDAC and chronic pancreatitis

| **Postoperative outcomes** | **Total (n=1,093)** | **USA (n=722)** | **NL (n=371)** | **P-value** |
| --- | --- | --- | --- | --- |
| Major complications  (Clavien–Dindo grade ≥3) | 407 (37.2%) | 192 (26.6%) | 215 (58.0%) | **<0.001** |
| POPF grade B/C | 238 (21.8%) | 89 (12.3%) | 149 (40.2%) | **<0.001** |
| PPH grade B/C  *Missing* | 95 (8.7%)  *1* | 59 (8.2%)  *1* | 36 (9.7%)  *0* | 0.398 |
| Bile leak grade B/C  *Missing* | 68 (6.2%)  *1* | 26 (3.6%)  *0* | 42 (11.4%)  *1* | **<0.001** |
| DGE grade B/C  *Missing* | 188 (17.4%)  *10* | 81 (11.2%)  *1* | 107 (29.6%)  *9* | **<0.001** |
| Primary DGE | 58 (5.4%) | 41 (5.7%) | 17 (4.7%) | 0.495 |
| Secondary DGE | 130 (12.0%) | 40 (5.5%) | 90 (24.9%) | **<0.001** |
| Hospital stay (days) | 8 (6–14) | 7 (6–11) | 12 (7–23) | **<0.001** |
| Readmission | 294 (26.9%) | 210 (29.1%) | 84 (22.6%) | **0.023** |
| Reoperation | 59 (5.4%) | 31 (4.3%) | 28 (7.5%) | **0.024** |
| In-hospital/30-day mortality | 15 (1.4%) | 6 (0.8%) | 9 (2.4%) | **0.032** |
| Use of the PORSCH algorithm | 371 (33.9%) | 0 (0.0%) | 371 (100.0%) | **<0.001** |

Legend supplementary file 7: Values are presented as medians with interquartile ranges (IQR) and frequencies with percentages (%). USA, United States of America; NL, the Netherlands; POPF, postoperative pancreatic fistula; PPH, postpancreatectomy hemorrhage; DGE, delayed gastric emptying.

# Supplementary file 8: Postoperative outcome in patients after the mastery learning curve

| **Postoperative outcomes** | **Total (n=1,424)** | **USA (n=1,174)** | **NL (n=250)** | **P-value** |
| --- | --- | --- | --- | --- |
| Major complications  (Clavien–Dindo grade ≥3) | 410 (28.8%) | 274 (23.3%) | 136 (54.4%) | **<0.001** |
| POPF grade B/C | 186 (13.1%) | 96 (8.2%) | 90 (36.0%) | **<0.001** |
| PPH grade B/C  *Missing* | 87 (6.1%)  *1* | 62 (5.3%)  *1* | 25 (10.0%)  *0* | **0.005** |
| Bile leak grade B/C  *Missing* | 39 (2.7%)  *1* | 19 (1.6%)  *0* | 20 (8.0%)  *1* | **<0.001** |
| DGE grade B/C  *Missing* | 182 (12.8%)  *6* | 123 (10.5%)  *4* | 59 (23.8%)  *2* | **<0.001** |
| Primary DGE | 80 (5.6%) | 72 (6.2%) | 8 (3.2%) | 0.069 |
| Secondary DGE | 102 (7.2%) | 51 (4.4%) | 51 (20.6%) | **<0.001** |
| Hospital stay (days) | 7 (6–11) | 7 (6–9) | 11 (7–21) | **<0.001** |
| Readmission | 350 (24.6%) | 289 (24.6%) | 61 (24.4%) | 0.942 |
| Reoperation | 56 (3.9%) | 41 (3.5%) | 15 (6.0%) | 0.064 |
| In-hospital/30-day mortality | 19 (1.3%) | 11 (0.9%) | 8 (3.2%) | **0.005** |
| Use of the PORSCH algorithm | 250 (17.6%) | 0 (0.0%) | 250 (100.0%) | **<0.001** |

Legend supplementary file 8: Values are presented as medians with interquartile ranges (IQR) and frequencies with percentages (%). USA, United States of America; NL, the Netherlands; POPF, postoperative pancreatic fistula; PPH, postpancreatectomy hemorrhage; DGE, delayed gastric emptying.
